# Understanding the nature and dimensions of litigation crowdfunding: A visual analytics approach

**DOI:** 10.1371/journal.pone.0250522

**Published:** 2021-04-27

**Authors:** Viju Raghupathi, Jie Ren, Wullianallur Raghupathi

**Affiliations:** 1 Koppelman School of Business, Brooklyn College of the City University of New York, Brooklyn, New York, United States of America; 2 Gabelli School of Business, Fordham University, New York, New York, United States of America; 3 Gabelli School of Business, Fordham University, New York, New York, United States of America; National Institute of Public Finance and Policy, INDIA

## Abstract

The escalating cost of civil litigation is leaving many defendants and plaintiffs unable to meet legal expenses such as attorney fees, court charges and others. This significantly impacts their ability to sue or defend themselves effectively. Related to this phenomenon is the ethics discussion around access to justice and crowdfunding. This article explores the dimensions that explain the phenomenon of litigation crowdfunding. Using data from CrowdJustice, a popular Internet fundraising platform used to assist in turning legal cases into publicly funded social cases, we study litigation crowdfunding through the lenses of the number of pledges, goal achievement, target amount, length of description, country, case category, and others. Overall, we see a higher number of cases seeking funding in the categories of human rights, environment, and judicial review. Meanwhile, the platform offers access to funding for other less prominent categories, such as voting rights, personal injury, intellectual property, and data & privacy. At the same time, donors are willing to donate more to cases related to health, politics, and public services. Also noteworthy is that while donors are willing to donate to education, animal welfare, data & privacy, and inquest-related cases, they are not willing to donate large sums to these causes. In terms of lawyer/law firm status, donors are more willing to donate to cases assisted by experienced lawyers. Furthermore, we also note that the higher the number of successful cases an attorney presents, the greater the amount raised. We analyzed valence, arousal, and dominance in case description and found they have a positive relationship with funds raised. Also, when a case description is updated on a crowdsourcing site, it ends up being more successful in funding—at least in the categories of health, immigration, and judicial review. This is not the case, however, for categories such as public service, human rights, and environment. Our research addresses whether litigation crowdfunding, in particular, levels the playing field in terms of opening up financing opportunities for those individuals who cannot afford the costs of litigation. While it may support social justice, ethical concerns with regards to the kinds of campaigns must also be addressed. Most of the ethical concerns center around issues relating to both the fundraisers and donors. Our findings have ethical and social justice implications for crowdfunding platform design.

## Introduction

Technological advancement has a marked impact on all categories of financing as well as funding sources. Online platforms (such as Kickstarter, GoFundMe, Crunchbase, Indiegogo and Rockethub) [1–3] have given rise to a novel method of funding called crowdfunding. This phenomenon impacts funding at both the industry as well as individual level. In this exploratory study, we applied visual analytics to study litigation crowdfunding, in which individuals raise funds to pay the legal costs of lawsuits [4, 5]. The study addresses the following key questions:

1. What are the dimensions of litigation crowdfunding; and
2. *What are the drivers of a successful litigation crowdfunding campaign*?

The increasing cost of litigation poses a challenge to the pursuit of justice [4–10] by creating a situation where defendants and plaintiffs are unable to afford attorney fees or other legal expenses either due to financial inability or difficulty in procuring loans from financial institutions [11]. Naturally, under such circumstances, people are less likely to contest their cases, which then results in either a guilty plea, no-contest plea, or self-representation [11]. Third party financing and contingency fee approaches do exist as alternatives to traditional litigation financing. However, there are drawbacks to each of the approaches. In third-party financing, the interest charges so high that it may put the borrower in debt [11]. In contingency fee approach, the outcome is uncertain, and in case of an award, it is likely that most of the award goes towards the attorney fees (based on the terms of the agreement). In this scenario, crowdfunding offers a viable funding source for people for their litigation cases [12]. Crowdfunding has been deployed as a source of funding for a variety of needs such as education, medical expenses, charities, and startup businesses. In the arena of litigation, it facilitates those pursuing litigation to procure funding from interested online communities.

Crowdfunding platforms enable litigants to have full control, within the realm of the platform norms [12], over the litigation process. This extends to appointing their own legal counsel, directing the course that the case can take, and avoiding unnecessary interference from funders. Norms can vary between different platforms. For example, in CrowdJustice, in order to be approved, litigants need to have retained an attorney prior to posting. This is to ensure that the platform can function without the risk associated with providing legal advice.

In order to illustrate the phenomenon of crowdfunding and its application in the legal arena, we present a background of prominent litigation campaigns around the world. In 2017, Maajid Nawaz, the chairman of a British think-tank, developed a crowdfunding campaign to fund a defamation action suit he filed against an advocacy organization for including his name as an anti-Muslim extremist. In 2019, a Scottish parliamentarian named Andy Wightman started a crowdfunding campaign to fight a defamation suit against him, filed by a wildlife protection organization regarding his blog post. This campaign, which raised about £60,000, was designed such that Wightman would reimburse the contributors pro rata if his defense turned out successful and he was able to recover his legal expenses. Another campaign by an Israeli journalist Igal Sarna to fund the defamation action against him, relating to his social media post about the Israeli Prime Minister Netanyahu and his wife, amassed above $45,000. In this campaign, any surplus funding and amounts reimbursed on appeal were pledged to go to the Association for Civil Rights. The above cases illustrate the relevance of litigation crowdfunding and its role in revolutionizing the civil justice process [5, 13, 14].

The phenomenon of litigation crowdfunding has the potential to promote justice by leveling the playing field. However, crowdfunding in general, and litigation crowdfunding in particular, have several ethical challenges surrounding their implementation. Crowdfunding studies have explored the conflict of self-interest and altruism in the context of ethics [15]. When this is explored in the specific domain of litigation crowdfunding, the conflict gets magnified–for example, is it ethical to fund cases that support societal or community causes (such as gender equality, racial equality), over others that support individual causes? Should cases geared with environmental goals have priority in funding over other cases? [16, 17].

The cost of litigation is yet another challenging factor in facilitating citizens access to the justice system. The issue of how much, and how often, to pay an attorney for legal representation, becomes a key element. Other ancillary costs include court fees, witness/expert remuneration, intangible harms and opportunity costs [4]. All of these can impact the ability to sue or defend a lawsuit. Costs can potentially inhibit justice in any of the following scenarios: when costs surpass the claimant’s benefits from litigation; the plaintiff lacks sufficient resources; or the disputant does not want to bear the cost of litigation due to uncertainty of the process [4, 5]. It is possible for investors in alternative litigation finance, such as crowdfunding, to manipulate litigation from the perspective of cost so that favorable discovery strategies or settlement options are discouraged. It is therefore incumbent that attorneys maintain independent judgment in response to these efforts. There have been certain ethical duties that the American Bar Association has proposed in a 2012 white paper, for lawyers in terms of imposing excessive fees, maintaining independent judgment, fee sharing with entities who are not lawyers, avoiding conflict of interest, confidentiality, and effects on settlement [18]. In this aspect, crowdfunding law firms are expected to include a disclaimer on not partnering or splitting fees with donors [11].

While several papers and studies have addressed these legal and social issues [4, 5], to our knowledge there is no empirical study that examines the dimensions of litigation crowdfunding. This study attempts to fill that gap by examining data from a popular litigation crowdfunding platform, CrowdJustice.com, to gain insight into the nature and dimensions of litigation crowdfunding, particularly in the ethical context. For example, we look at the types of cases (projects) most likely to be funded. Addressing these types of questions from an ethical perspective will have major policy implications, impacting voluntary ethical compliance and regulatory changes.

The rest of the paper is organized as follows: the next section provides background information on crowdfunding, particularly litigation crowdfunding. An outline of our methodology follows. Then, the results of visual analytics are presented, along with the scope and limitations of the study. Finally, implications and future research are covered, followed by conclusions.

## Background

Being a relatively new phenomenon, litigation crowdfunding is considered to be in a nascent stage. The principle lies in relying on small donations to offset huge litigation costs [4, 5, 10]. In litigation crowdfunding, a large number of individuals (the crowd) who are willing to donate, represent the financial backer. Instead of investing a large sum of money toward the litigation, most of these individuals donate small amounts. The phenomenon presents the potential to mitigate financial concerns surrounding funder control and possible conflicts of interest [4, 5, 11].

Crowdfunding models can be investment-based or non-investment based [4, 5]. In investment-based models, the person who contributes expects financial return which can take the form of a share in the claimant’s future gain (as in equity-based crowdfunding) or repayment with interest (as in debt-based crowdfunding). In these models, funders support cases that they expect to be successful and that can bring them profits. On the other hand, in non-investment based crowdfunding models, the person who contributes may expect either a non-monetary benefit (as in reward-based crowdfunding) or nothing in return (as in donation-based crowdfunding). In donation-based crowdfunding, the funders are increasingly driven by altruism or empathy towards a case [4]. Litigants can choose from a variety of dedicated crowdfunding platforms to finance their legal proceedings. Some platforms such as LexShares in the U.S., and AxiaFunder in the U.K, are both investment-based platforms that select cases that have strong merit and high likelihood of success. CrowdJustice, on the other hand, is a donation-based platform where people support cases without any expectation of monetary or non-monetary reward [4, 5].

Regardless of the model, the phenomenon of crowdfunding generally involves a *fund seeker* who seeks funding for an idea or a project, and the *crowd* (funder) or the people who contribute funds to the idea or project. In most cases, the connection between fund seeker and funder is made possible via an intermediate, online crowdfunding *platform* [4, 5]. In addition to acting as a matchmaker, the platform promotes and publicizes the projects and endeavors to draw the largest number of potential investors. To this end, the platform builds and maintains a sophisticated presence, operating online to take advantage of technology innovation. The platform also applies due diligence activities that include combination of background checks, credit checks, account monitoring, site visits, cross-checks, and third-party proof [19–21]. Due diligence is more pronounced in larger crowdfunding platforms, and for equity and lending crowdfunding platforms [19]. Updates to crowdfunding legislation can facilitate creating a positive impact on the application of platform due diligence [19]. Due diligence can help avoid fraudulent campaigns from being posted [20] on the platform and can mitigate information asymmetries between the fund seeker and the funder [19]. Due diligence application can be a facilitator for fundraising campaign success. It can also enable increasing the investor base for a campaign or the total amount raised on a platform [19–21]. Applying due diligence can be a costly endeavor and therefore platforms have to evaluate if they receive sufficient benefits to cover the expense.

In exchange for its involvement, the crowdfunding platform routinely takes a percentage of the proceeds, often referred to as a success fee. The amount of this fee varies from 4% to 9% of the raised capital, depending on the crowdfunding campaign model [1–3]. Along these lines, fund seekers normally set a fundraising goal to signal their aspirations as well as the feasibility of the project in terms of the potential risk for investors. The goal also helps assess parameters for calculating the allocation between the investors’ reward and the platform fees for the campaign. This type of funding is particularly relevant to cases in public interest litigation, since funding and support are limited and contingent on the charitable time contribution of attorneys and other supporters [1–3, 4, 5].

Litigants can deploy six alternative methods to pay for litigation: third-party litigation funding, unbundled legal services, legal expense insurance, crowdfunding, alternative fee arrangements, and publicly-funded litigation funds [22, 23]. Litigation crowdfunding combines third-party funding (TPF) with crowdfunding. It offers an online venue for litigants to finance a part or all of their civil dispute costs through multiple donors making small contributions. Litigation crowdfunding platforms can be general platforms, specialized platforms or traditional business/personal websites.

Some of the earliest crowdfunded litigation campaigns were launched through general crowdfunding platforms such as Indiegogo [4, 5]. The first specialized crowdfunding platform to focus on crowdfunding of commercial lawsuits, including intellectual property violations, business torts, and professional negligence, and excluding personal injury claims, was LexShares—established in 2014 in the United States. As a platform, LexShares is subject to Securities and Exchange Commission regulation [4, 24]. It retains a percentage of the funds raised and takes a part of the investors’ profit if the claim succeeds [24].

Fundrazr, a platform established a few weeks after LexShares, differs from LexShares [25] in that it does not require investors to be SEC-accredited [24]. Yet another platform, CrowdJustice is based in United Kingdom [26]. Both Fundrazr and CrowdJustice retain a percentage of funds raised (7% and 5%, respectively) (https://www.crowdjustice.com/how-it-works/). Neither has, to date, funded tort litigants.

Considering the various models, the impact that crowdfunding has on litigation funding is stronger than that of commercial TPF [5]. For one, crowdfunding is available to people pursuing nonmonetary remedies (e.g., injunction or declaratory judgment) [5]; second, TPF is only available to claimants who can offer collateral for a loan (https://www.lexshares.com/litigation_finance_101). Potential defendants can attempt to get insurance based on likely forecast of the legal expenses. Generally, defendants also cannot obtain special financial support after the event has occurred and harm done due to the event [5]. On the other hand, donation- and reward-based crowdfunding can be available to defendants, as demonstrated in the Wightman and Sarna cases [5]. Lastly, unlike traditional TPF which covers process costs, crowdfunding is also available for outcome costs, as evidenced in the Sarna case [5].

Crowdfunding helps litigants in several ways [4, 5] including making it possible to proceed with a litigation project, in the first place. The feature of high litigation costs can impede justice, as the process necessitates bearing significant costs with no guarantee of outcome. This can make it difficult for risk-averse people or for those facing financial difficulty. Ultimately, only those who have deep pockets and/or feel extremely aggrieved, would pursue the litigation process [5]. In this scenario, investment-based crowdfunding or TPF can alleviate the financial obstacles and enable access to justice [27]. However, both of these modes are not available in a situation when litigation costs exceed the expected benefits since the benefits represent the upper boundaries of the funders’ return [5].

In this research, we also address how case descriptions can impact the funding success or failure of the case, in the context of litigation crowdfunding. We examine, with the limited data availability, the valence, arousal, and dominance (VAD) constructs from the descriptions. Studies on affect highlight that the factors of valence, arousal and dominance (VAD) have the potential to surface out of an individual’s interest, level of activation, and/or perceived level of control for a particular situation, through textual communication (e-mail), narrative descriptions (product descriptions, book review/synopsis), and case descriptions in crowdfunding platforms [28–39]. Emotions and moods (i.e., joy, anger, sadness) are pervasive in the daily activities of individuals. They act as the driving forces when people are dealing with a variety of activities such as purchases, daily chores, investments, or charitable contributions [34, 40]. For example, [41] and [42] adopted the VAD affect model to depict an individual’s emotional range (note that affect and emotion are used interchangeably in earlier research). Along these lines, we evaluate the impact of emotions of valence, arousal, and dominance in case descriptions in terms of the success or failure of crowdfunding litigation cases.

The literature on litigation financing, particularly crowdfunding, is also at a nascent stage. As mentioned, much of the published work has focused on legal and strategic aspects [4, 5, 10, 22]. While numerous studies have looked at crowdfunding in other domains, there are no empirical studies on the causes, success factors, and dimensions in litigation crowdfunding. Gaining insight into these aspects is critical from an ethical and social justice perspective. Our exploratory research addresses the paucity and offers a panoramic insight into the current state of litigation crowdfunding. It explores the dimensions of litigation crowdfunding as well as the factors influencing successful campaigns.

This research makes at least three key contributions to existing conceptual literature. First, it provides an empirical understanding of the nature of litigation crowdfunding. Second, it sheds light on the various dimensions of litigation crowdfunding. And third, from a policy perspective, the study contributes to the understanding of the balance of ethics and justice in litigation crowdfunding. In this instance, crowdfunding platforms may consider ethical, legal, moral, and justice aspects in their design and business model.

## Methods

### Visual analytics

We adopt a data-driven visual analytics approach utilizing descriptive analytics [43–45] to get insight into the phenomenon of litigation crowdfunding. We use data of litigation cases from the litigation crowdfunding platform CrowdJustice.com. Visual analytics enables effectively analyzing and comprehending large datasets in real time [45, 46]. By integrating the capabilities of the computer with human expertise, it allows exploring unexpected patterns and insights that can then present novel solutions [47, 48]. It mitigates information overload by converting information into viable opportunities and allowing researchers to holistically examine the results along with the processes leading to the results [45, 46, 48]. The objective is story telling through visualization, statistical analysis and data mining [45, 47]. Compared to other models, descriptive analytics tends to be more data driven, focusing on describing the data ‘as is’ with no preconceived assumptions. It facilitates comprehending past and current patterns and trends and utilizing the same for informed decision making [44, 47, 48]. Information is depicted graphically through charts and images, using the processes of categorization, characterization, and aggregation [47, 48].

We draw from the literature on crowdfunding and litigation crowdfunding, in particular web-based platforms, and empirically explore the following key questions, among others:

1. *What are the different categories of cases*, *and what is the distribution among these categories*?
2. *Which categories (cases) have raised the highest average amount*?
3. *What is the proportion of successful (funding goal achieved) v*. *unsuccessful (funding goal not achieved) cases*? *How does this reflect in terms of the categories*?
4. *Which law firms (lawyers) have the highest number of successful crowdfunding cases*?
5. *What is the association*, *if any*, *between successful cases and amount raised in different categories*?
6. *Does updating a case description increase the likelihood of successful funding*? *If true*, *which categories are more successful*?

### Data collection and variables

The data source for this study is the crowdfunding platform *Crowdjustice*.*com*, founded in the United Kingdom in 2014. This commercial crowdfunding platform, aimed at improving access to the legal system, enables individuals and organizations to raise money specifically for legal cases in the U.S and U.K. The platform evaluates each campaign to ensure that it engages a qualified attorney and that all funds go to the trust account of the attorney’s client. If a campaign does not meet its funding goal, the donors are not charged. In 2015, CrowdJustice announced that it would take a commission of 5% from the cases it funds.

We collected data in early April 2019 from the CrowdJustice.com website. We crawled the website using the packages Selenium and Beautiful Soup packages in Python. Our data collection method complied with the terms of conditions of the CrowdJustice website. Our methodology includes the stages of data collection and variable selection, data preparation, analytics platform and tool selection, and analytics implementation (Table 1).

**Table 1:** Methods.

**Data Source**
CrowdJustice.com
**Data Preparation**
Data crawled from CrowdJustice.com website in csv format and prepared for loading into a BI tool
**Analytics Platform/Tools Selection**
Tableau and Python

We extracted 565 records of crowdfunding litigation projects that had successful funding. Our dependent variable is ‘goal_achieved’, which indicates that a case obtained successful funding (defined as either reaching or exceeding its funding goal).

The independent variables cover project data (number of pledges, location, and category), tone of the case description (overall sentiment score), emotional sentiments of the fund seekers from the case description (valence, arousal, and dominance), and wordings in the case description (number of words, ratio of misspelt words, and number of difficult words). Table 2 shows the details of each variable in terms of its definition as well as whether it was directly available on the website or was calculated.

**Table 2:** Variables used in the study.

Variable Name	Description	Available or Calculated
Case name	The title of the case	Available
Category	The category type of the legal case	Available
Pledge	Number of pledges	Calculated
Status	The status of the case (Closed / Funded)	Available
Target	The amount needed	Available
Raised amount	The amount raised	Available
Lawyer	The name of the law firm	Available
City, State, Country	The geographic location of the case	Available
Tone	Sentiment score of the description	Calculated
Length_misspelled	Number of misspelled words in description	Calculated
Update	Whether the description was updated (True / False)	Available
Percentage_of_raised	Raised amount as percentage of target amount	Available
Goal_Achieve	Whether the project succeeded (Failed / Successful) in achieving the target	Available
Desc_v	The weighted average score of valence in description	Calculated
Desc_a	The weighted average score of arousal in description	Calculated
Desc_d	The weighted average score of dominance in description	Calculated
Len_desc	Number of words in the case description	Calculated
Misspell_ratio	Number of misspelt words as a ratio of total number of words in description	Calculated
Difficult_words	Number of difficult words in a description	Calculated

The weighted average scores for valence, arousal, and dominance in case description were calculated using the sentiment word dictionary defined in [49]. The dictionary has weights assigned to every emotional word in the three dimensions of valence, arousal, and dominance based on a large-scale survey. For each case, we extracted the emotional words from the description and aggregated the weighted average scores of valence, arousal, and dominance based on the weights of these words along the three dimensions.

The tone is calculated as the difference between the number of positive and negative words in the description, divided by the description length (number of words). If the tones are mostly positive, it indicates that there are more positive words in the description. The ratio of misspelt words in the description is calculated as the number of misspelt words, divided by the length of the description (total number of words). The readability of the case descriptions was assessed using the number of difficult words included. To measure the number of difficult words, we adapted the Fog Index (also known as the Gunning-Fog Index), from the computational linguistics literature [50], which captures the complexity of text as a function of syllables per word and words per sentence. Complex words are defined as those words with three syllables or more. We adopted this measure in our research to assess the difficulty of the description and the effect on success or failure in funding.

This study used Python to process data, including data cleaning, adding or removing variables, and computing calculated fields of the variables. Then, for the data analysis, we used Tableau, an advanced business intelligence and data analysis tool for visualization and descriptive analysis. Approaches for analysis include ranking, association, and comparison of the crowdfunding data. We will discuss the results of our analyses in the following section.

## Results and analyses

**Fig 1:**
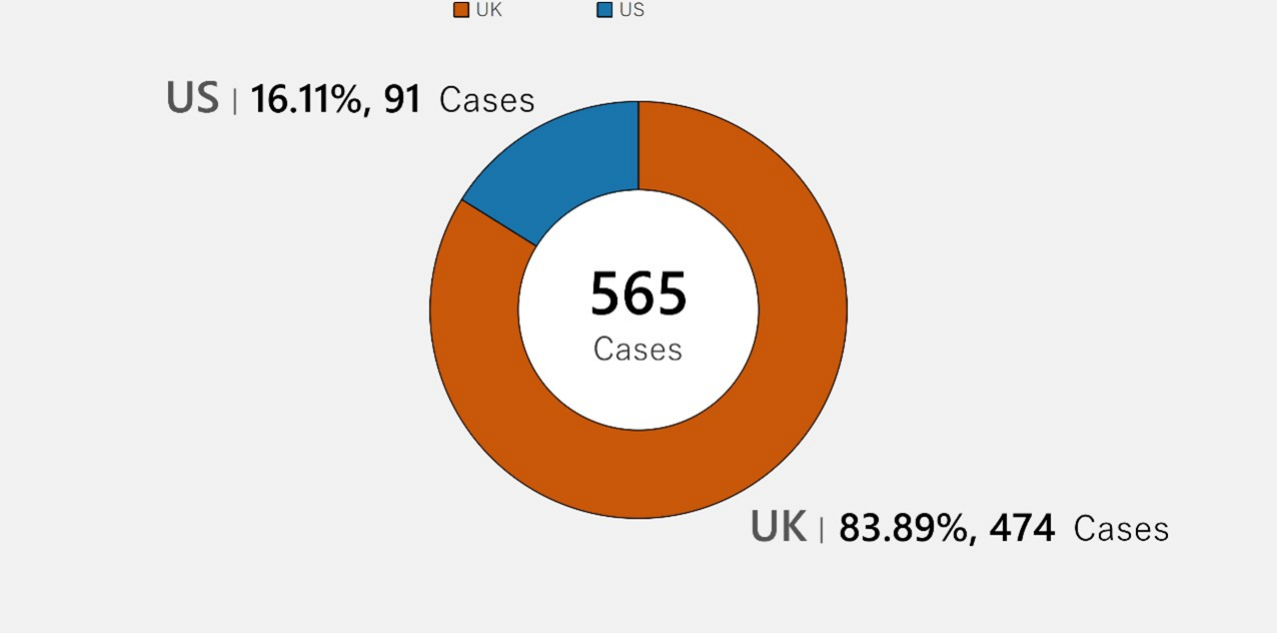
Distribution of cases.

Fig 1 shows the distribution of our sample of litigation crowdfunding cases on the platform CrowdJustice.com. The total number of litigation crowdfunding cases is 565, distributed between the U.K, with 474 (about 84%), and the U.S., with 91 cases (about 16%).

**Fig 2:**
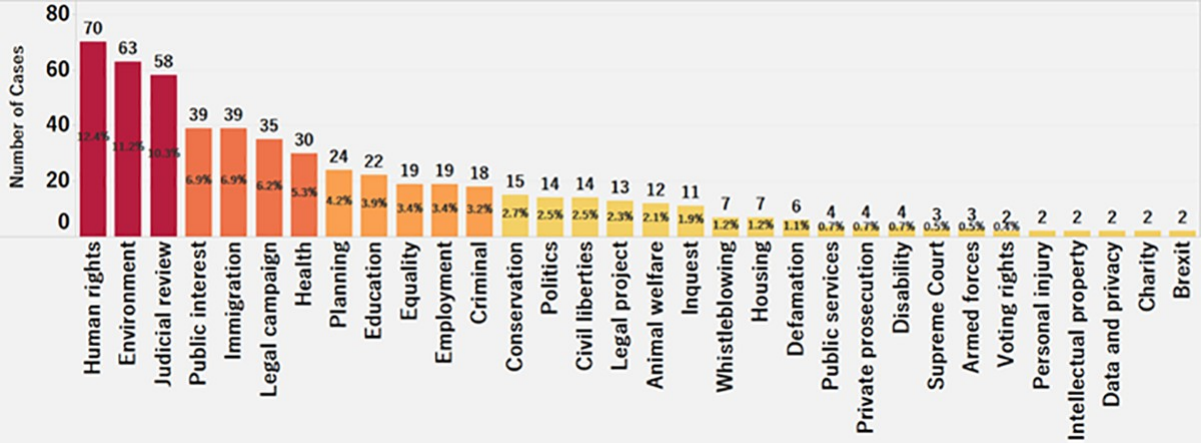
Distribution of cases by category.

Fig 2 shows the distribution of the crowdfunding cases across different categories. The number of human rights cases, at 70 (about 12%), is the highest; followed by environment, with 63 cases (about 11%); and judicial review, with 58 cases (about 10%). It would appear that individuals who are involved in litigation regarding human rights, judicial review, environment, immigration, and public interest are more likely to seek crowdfunding. The findings project that crowdfunding platforms should focus their efforts to promote the phenomenon in other less successful categories, such as voting rights, personal injury, intellectual property, and data & privacy.

**Fig 3:**
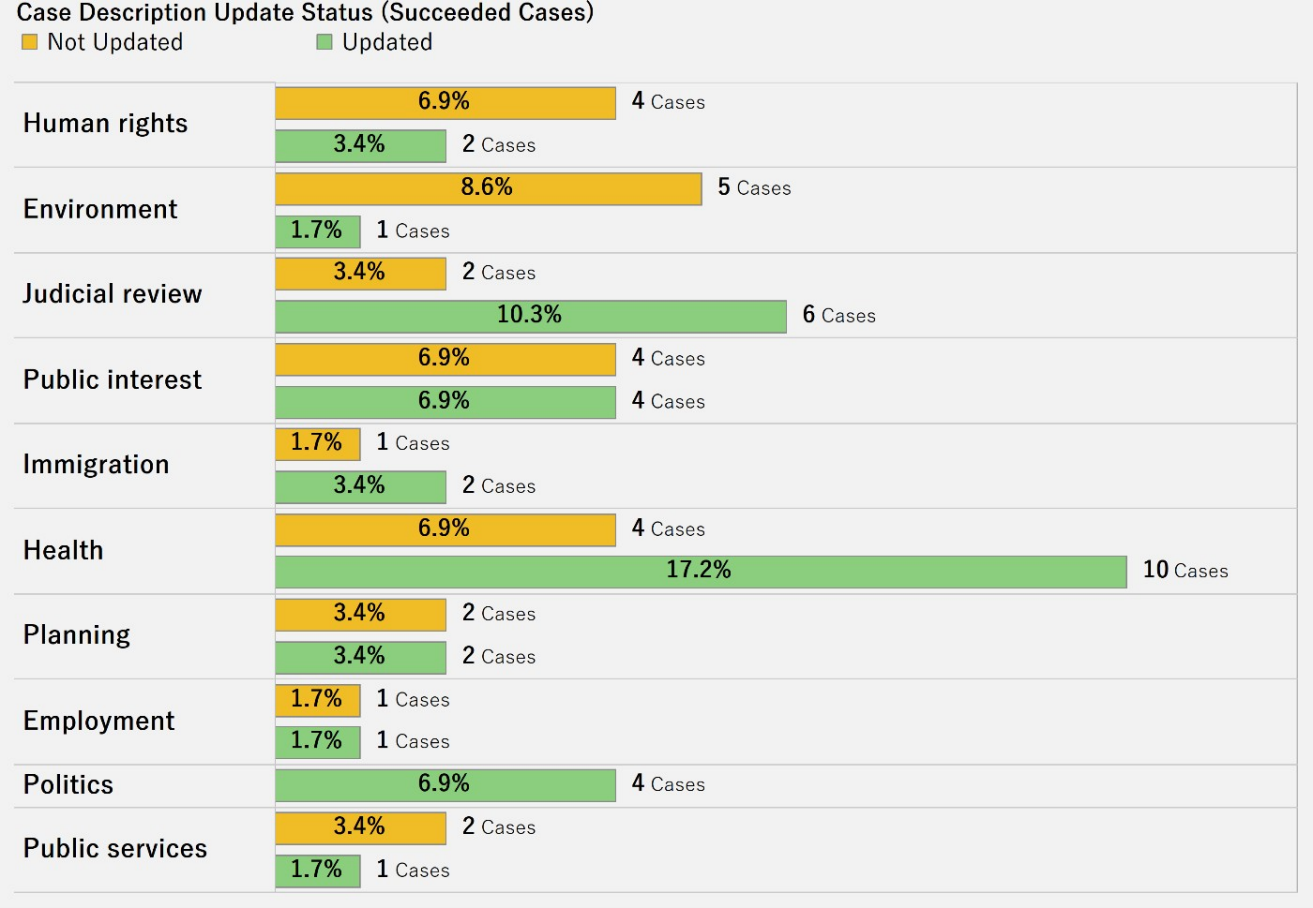
Distribution of successful cases by category and update status.

Fig 3 depicts the distribution of successful cases across different legal categories, broken down by the update status of the case description. The chart shows whether updating a case description increases the likelihood of funding success for the case. Indeed, when case descriptions are updated there are more successful cases in health, immigration, and judicial review categories. This is not the case, however, for categories such as public service, human rights, and environment. There appears to be no consistent trend in the relationship between update and number of successful cases. Not all the successful cases involved an updated description.

**Fig 4:**
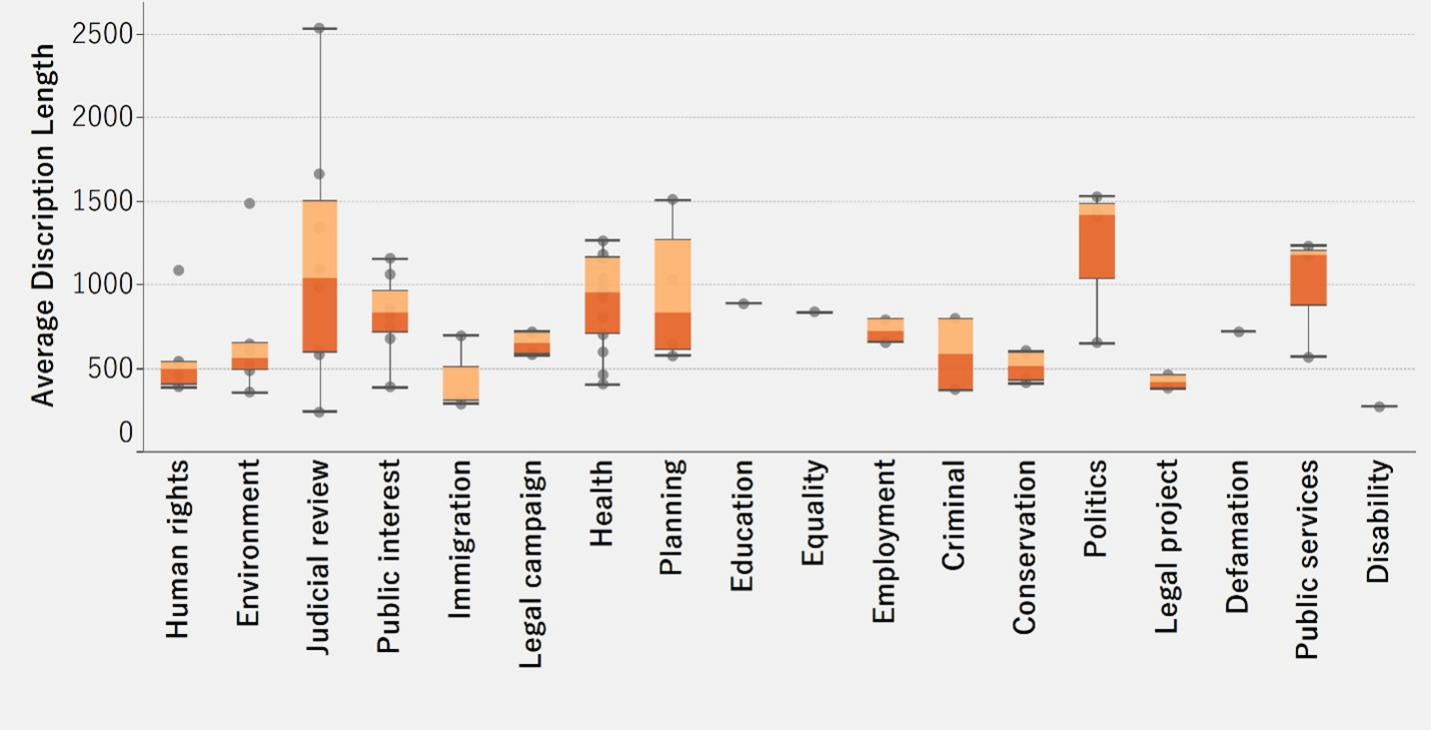
Distribution of successful cases by category and average description length.

Fig 4 shows a box plot representing the distribution of the average description length for successfully funded cases across different legal categories. The box plot depicts the summary information for the lower quartile (lowest 25% of cases), the middle 50% of cases (shown in the box with the median divider), and the upper quartile (highest 25% of cases). In the box containing the middle 50% of the cases, the darker color depicts cases that have an average description length lower than the median, and the lighter color depicts cases with an average description length above the median. Among all the categories, judicial review has the highest average description length (2532 words) and the widest range (about 2294). The symmetrical coloring of the box indicates that out of the middle 50%, there is an equal spread of cases with description lengths above and below the median (1038 words). On the other hand, for politics and public services categories, the data are skewed with most cases having an average length below the median as indicated by the darker color (1432 for politics and 1174 for public services). For disability, the box being collapsed indicates low to no variability in average description length (270 words), which is the lowest. However, people are likely to donate to litigation crowdfunding cases in disability, regardless of the length of the case description.

**Fig 5:**
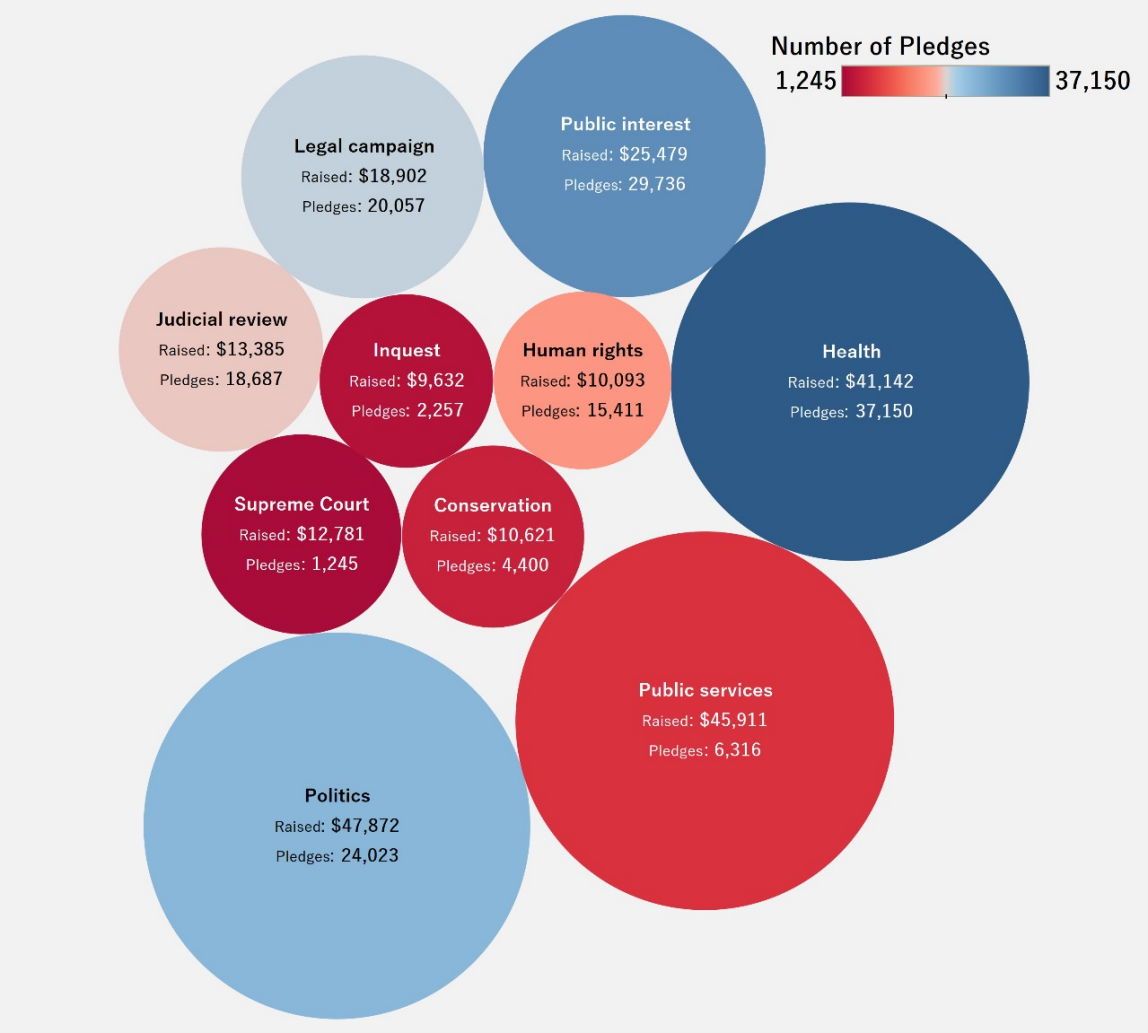
Top 10 categories by average raised amount.

Fig 5 is a bubble chart that shows the top ten major categories for litigation crowdfunding cases in terms of highest average amount raised. The size of the bubbles represents the average raised amount, and the color of the bubbles represents the number of pledges for cases in the categories. The bubbles are also labeled with the average raised amount for cases in each category and the total number of pledges. The public is willing to donate more money to cases in health, politics and public services.

**Fig 6:**
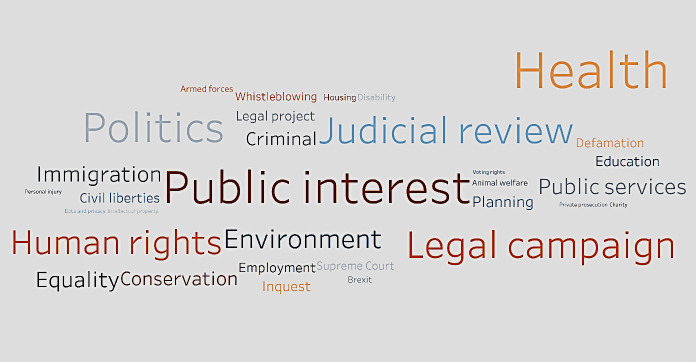
Word cloud of popular crowdfunding categories.

Fig 6 is a word cloud that shows the popular crowdfunding categories in terms of the number of pledges. The size of the font represents the number of pledges. The bigger the font, the more the number of pledges in the category. Pledges pay more attention to cases in public interest, health, and legal campaigns, resulting in more likelihood of raising additional funds.

**Fig 7:**
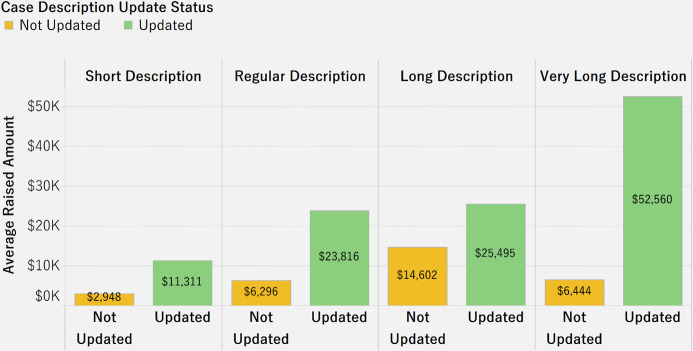
Association of average raised amount with description length & update status.

We explored the relationship of average amount raised with the update status and the length of the case description (Fig 7). We categorized the case descriptions with length less than 100 words as very short, those with 100 to 500 words as short, those with 500 to 1000 words as regular, those with 1000 to 1500 words as long, and those over 1500 words as very long. According to the chart, when the case descriptions are updated with more details, the cases tend to raise greater amounts. Donors prefer to spend more money on updated cases with longer descriptions. Donors may believe that lengthy descriptions imply a more reliable source and a more serious case.

**Fig 8:**
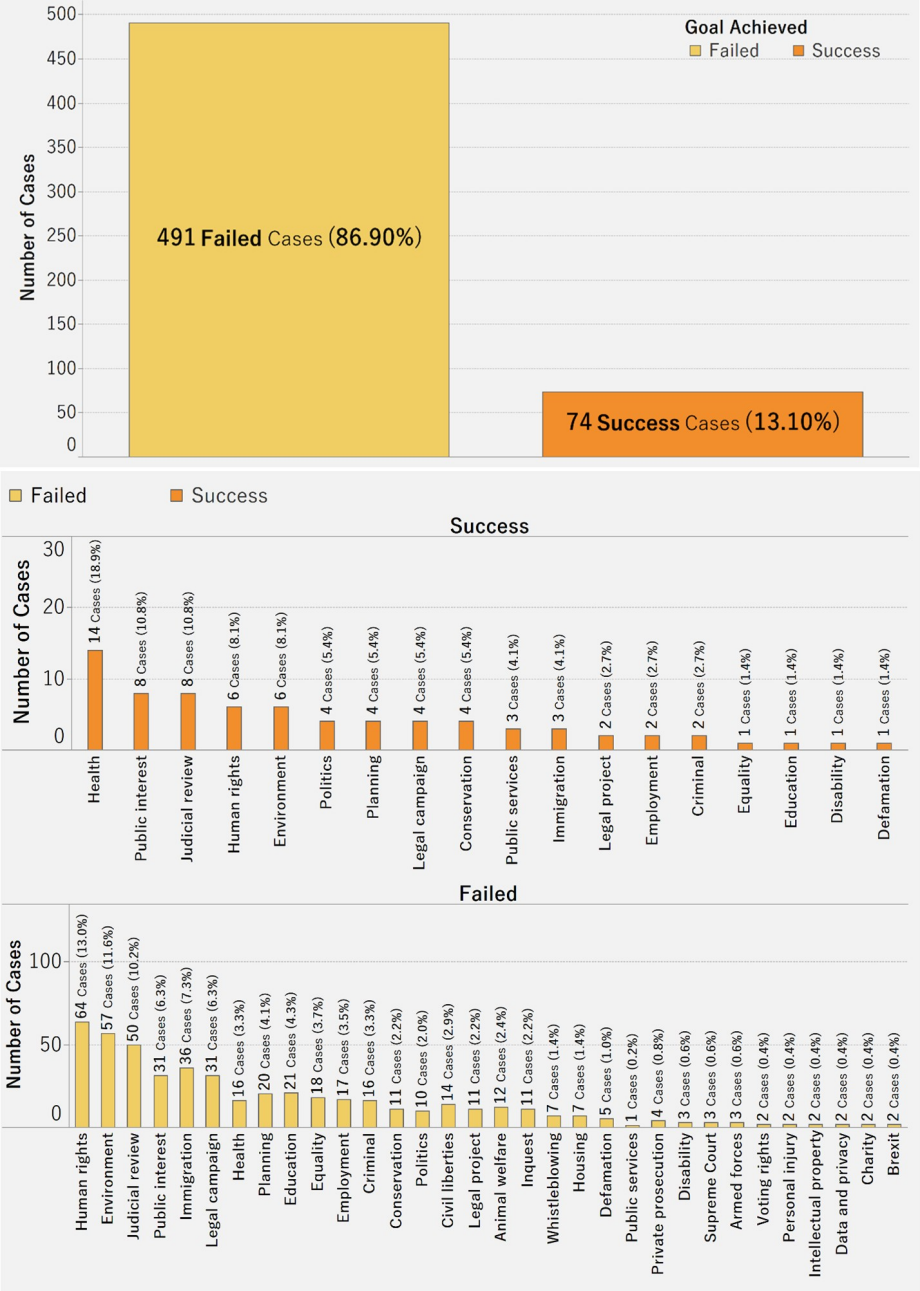
a. Distribution of failed and successful crowdfunding cases. b. Distribution of failed and successful crowdfunding cases across category.

Fig 8A presents the number of failed and successful crowdfunding cases (in terms of amount raised). The number of failed cases is more than six times the number of success cases. It appears that it is difficult for crowdfunding projects to achieve the funding goal. This highlights the need for fundraising platforms to discern possible reasons for the high failure rate.

We then explored the number of failed and successful crowdfunding cases (in terms of amount raised) by category (Fig 8B). Health has the highest number of successful cases (about 19%), while Human Rights has the highest number of failed cases (about 13%).

**Fig 9:**
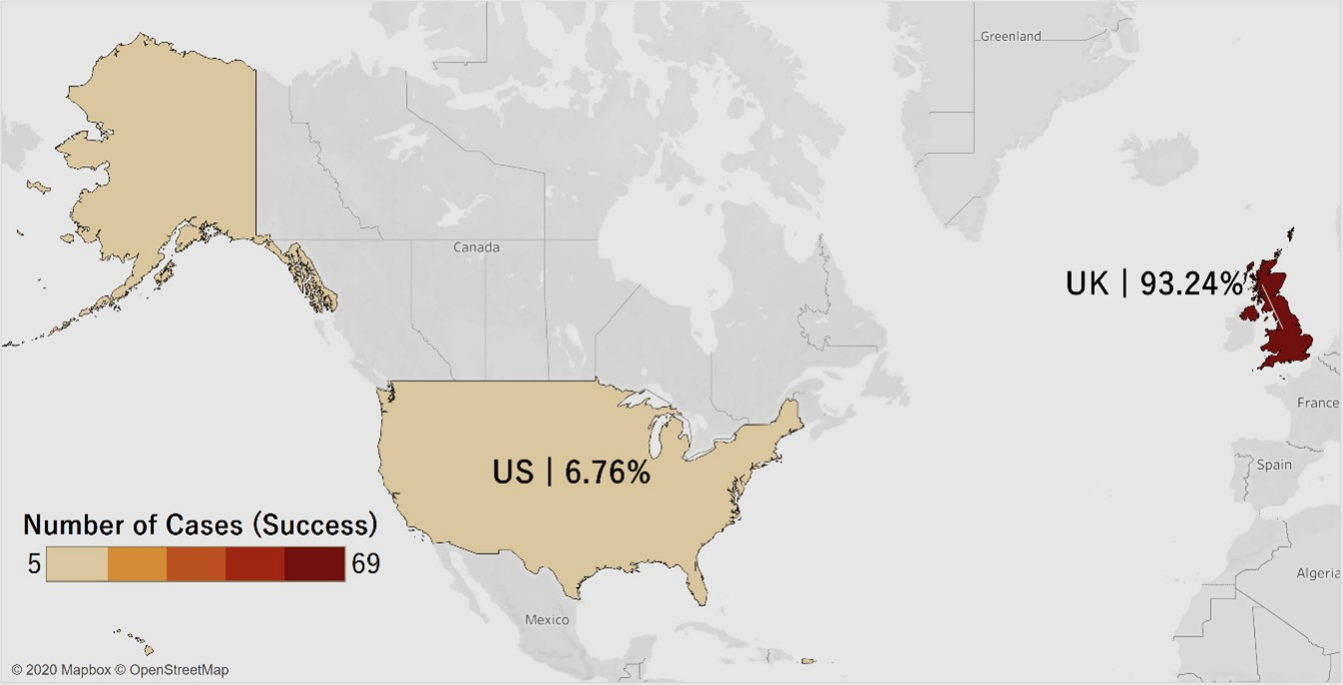
Distribution of successful crowdfunding projects in U.K. and U.S. Visualization Source: Tableau.

Fig 9 presents the number and percentage of successful cases in the U.K. and the U.S. There are many more successful cases in the U.K. (about 93%) than in the U.S (about 7%). It is possible that the phenomenon of litigation crowdfunding in the U.K may be more advanced, considering it originated there. Also, since the data source is a U.K.-based platform, it is more likely there are more cases in the U.K. overall compared to the U.S.

**Fig 10:**
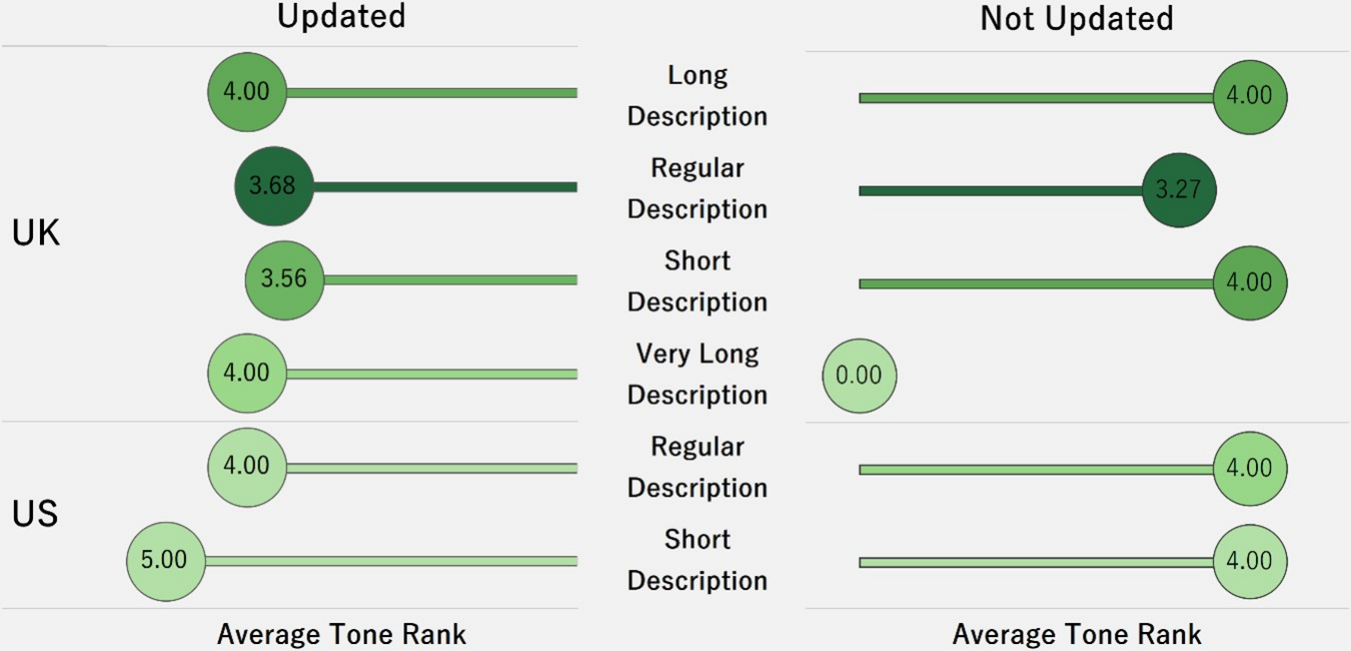
Tone rank for successful cases based on length of description and update.

Fig 10 is a dot plot that shows the average sentiment score (tone) for different case description lengths in the U.K. and the U.S. The tone is calculated by the difference between the number of positive and negative words in a case description, divided by the description length. If the tones are mostly positive, it indicates that there are more positive words in the description. After updating the description, the tone rank is higher, and the number of successful cases is also greater for both the U.K. and the U.S. However, there is insufficient information to suggest that the length of description influences the tone rank.

**Fig 11:**
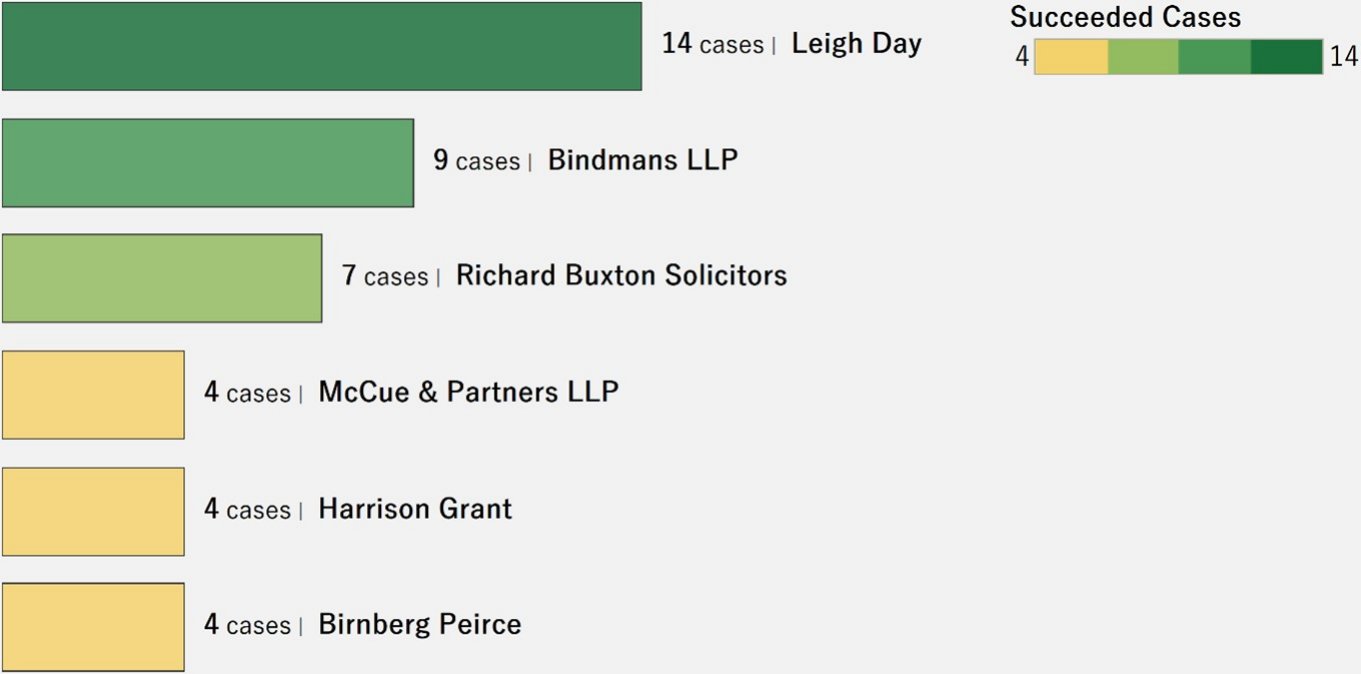
Distribution of successful cases by lawyer/law firm.

Fig 11 shows a ranking of the top six lawyers/law firms in terms of number of successful crowdfunding cases. According to this chart, Leigh Day, based in the U.K., is ranked as the top firm, with the highest number (14) of successful cases. Funders attribute reputable law firms with more credibility in seeking funding and are more willing to contribute to these. Future research should investigate the role of organizational variables, such as reputation, and its influence on the amount raised for the cases.

**Fig 12:**
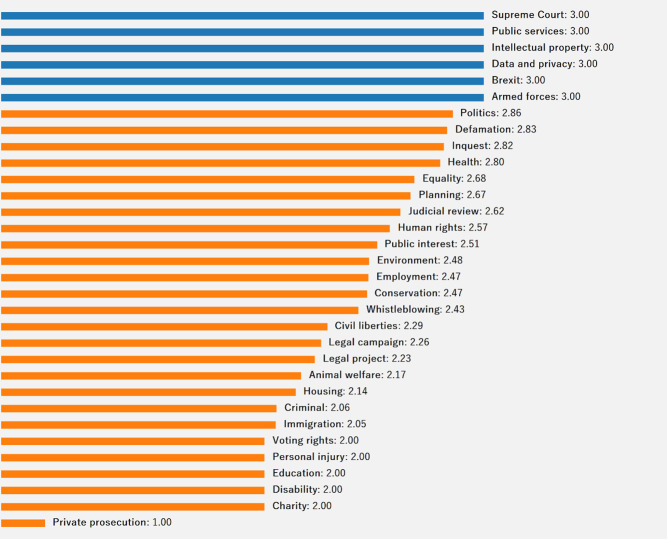
Average misspelled words in different categories.

Fig 12 shows the distribution of the average number of misspelled words in case descriptions for the different categories. The highest average misspelled words in a case description is approximately three words. The categories with the highest average misspelled words are Armed Forces, Brexit, Data and Privacy, Intellectual Property, Public Services, and the Supreme Court. On average, private prosecution has the lowest number. This data point suggests that those seeking funding should focus on the grammatical accuracy of writing in the case descriptions.

**Fig 13:**
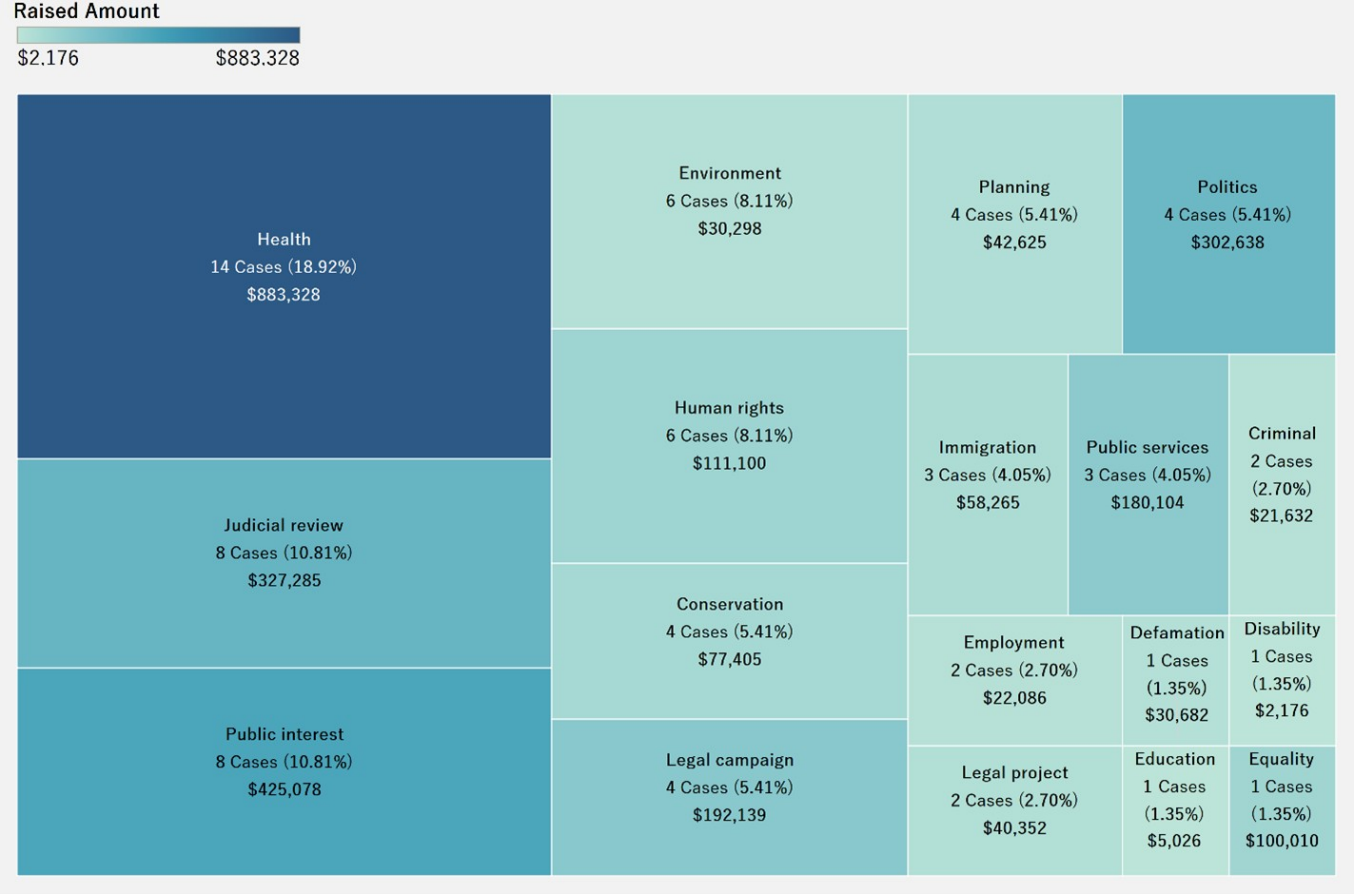
Association Between successful cases and amount raised by category.

Fig 13 is a tree map that shows the number and percentage of successful cases, as well as the amount raised in different categories. The size represents the number of cases, and the color intensity represents the raised amount. Health accounted for the greatest number of successful crowdfunding cases as well as for the highest amount raised. Donors generally believe that health is important to life. Therefore, when they perceive fund seekers to be struggling with health-related litigation cases, they are more likely to fund the cases. Furthermore, we also see that the greater the number of successful cases, the higher is the amount raised.

**Fig 14:**
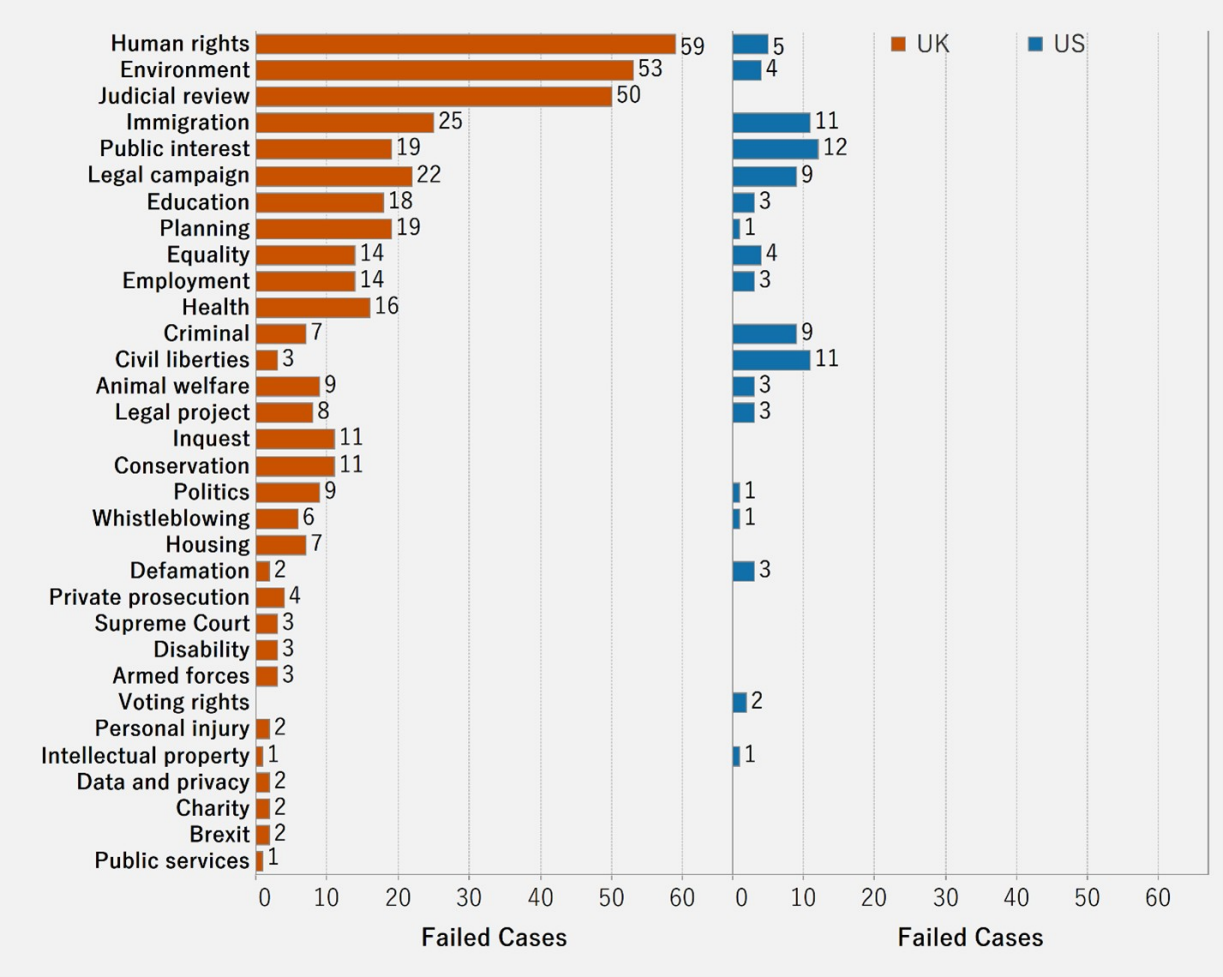
Distribution of failed cases by category and country.

Fig 14 shows the distribution of failed cases across categories and countries. In the U.K., the highest number of failed cases is in the category of human rights, while in U.S it is in the category of public interest. The number of failed cases does not imply that pledges in the U.K. and the U.S. are unwilling to donate to human rights and public interest cases but rather that the competition among the crowdfunding cases in human rights and public interest is likely intense, resulting in a higher number of failed cases.

**Fig 15:**
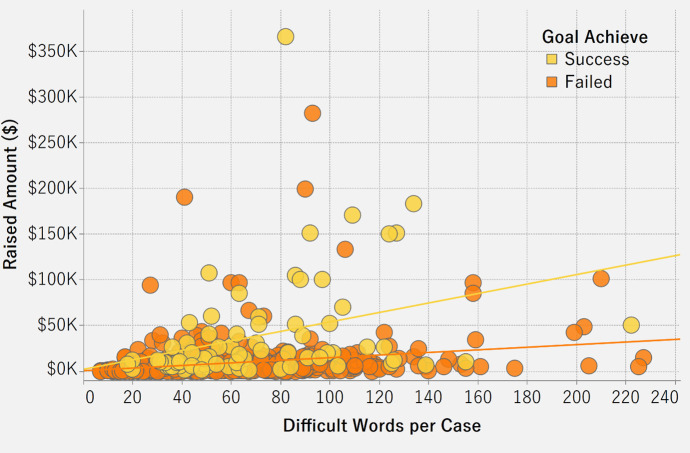
Association between amount raised and difficult words in case description.

Fig 15 is a scatter plot that shows the linear relationship between amount raised and the number of difficult words in a case. The number of difficult words in a description is a significant predictor of raised amount for failed (p<0.0001) and successful cases (p<0.01). Whether the funding goal is achieved (success) or not (failure), the relationship between the number of difficult words and the raised amount is always positive. Both R-square values are low. For failed cases, approximately 5% (R^2^ = 0.0497515) of the variation in the amount raised can be explained by the number of difficult words. For successful cases, around 10% (R^2^ = 0.107085) of the variation in the amount raised can be explained by the number of difficult words. As the number of difficult words in a case’s description increases, the raised amount also usually increases. However, it needs to be acknowledged that our sample consists of only 565 cases. Therefore, a broader conclusion can be reached only with more data.

**Fig 16:**
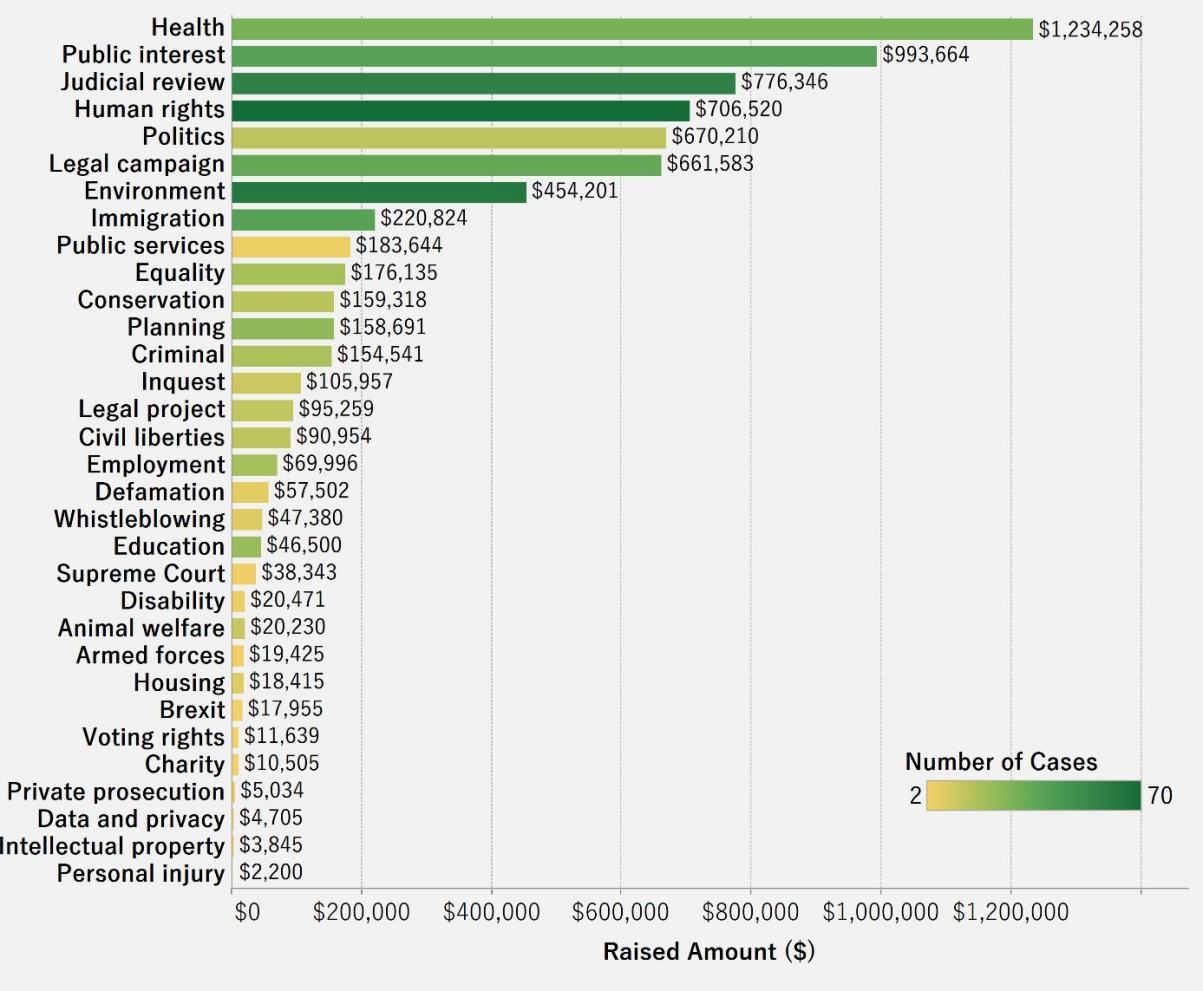
Raised amount and number of cases by category.

Fig 16 shows the total amount raised as well as the number of cases for each category. The intensity of the color in the bar represents the number of cases for the category. We can see that health-related legal crowdfunding cases have the highest amounts raised, followed by public interest. In health, despite the fact that the number of cases is not high, the amount raised is large. For cases related to the environment, the number of cases is high, but the amount raised is relatively lower than those in the categories of health and public interest. Health and public interest cases are most likely to succeed.

**Fig 17:**
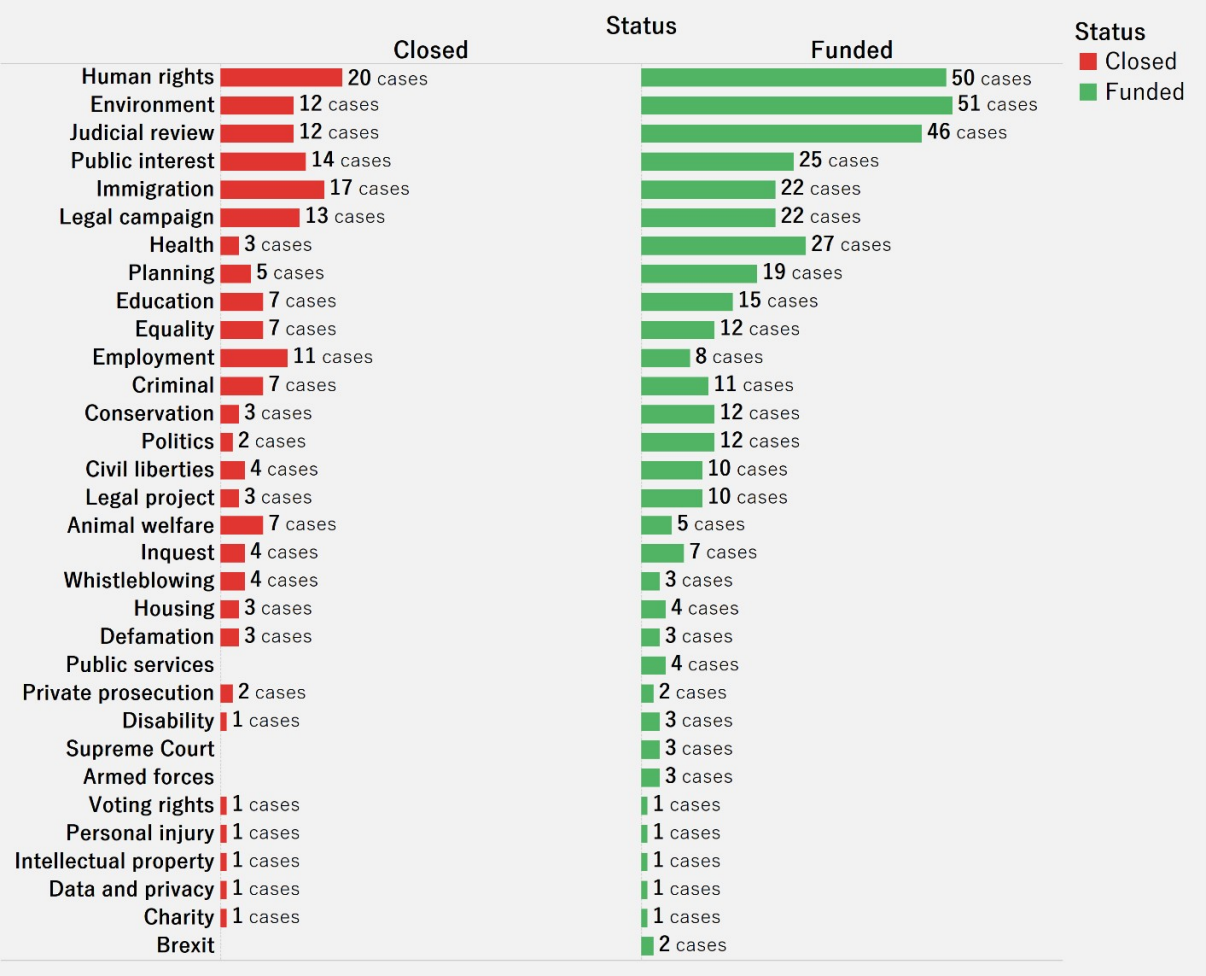
Bar chart for number of cases in categories by status.

Fig 17 shows the number of cases by category and by status (whether the case is closed or funded). Most of the categories are funded rather than closed. Funded crowdfunding cases are more than the double of closed cases for human rights, environment, and judicial review. However, funded cases do not necessarily imply that the raised amount exceeds the target amount. Further analysis is required to investigate the success rate for each category.

**Fig 18:**
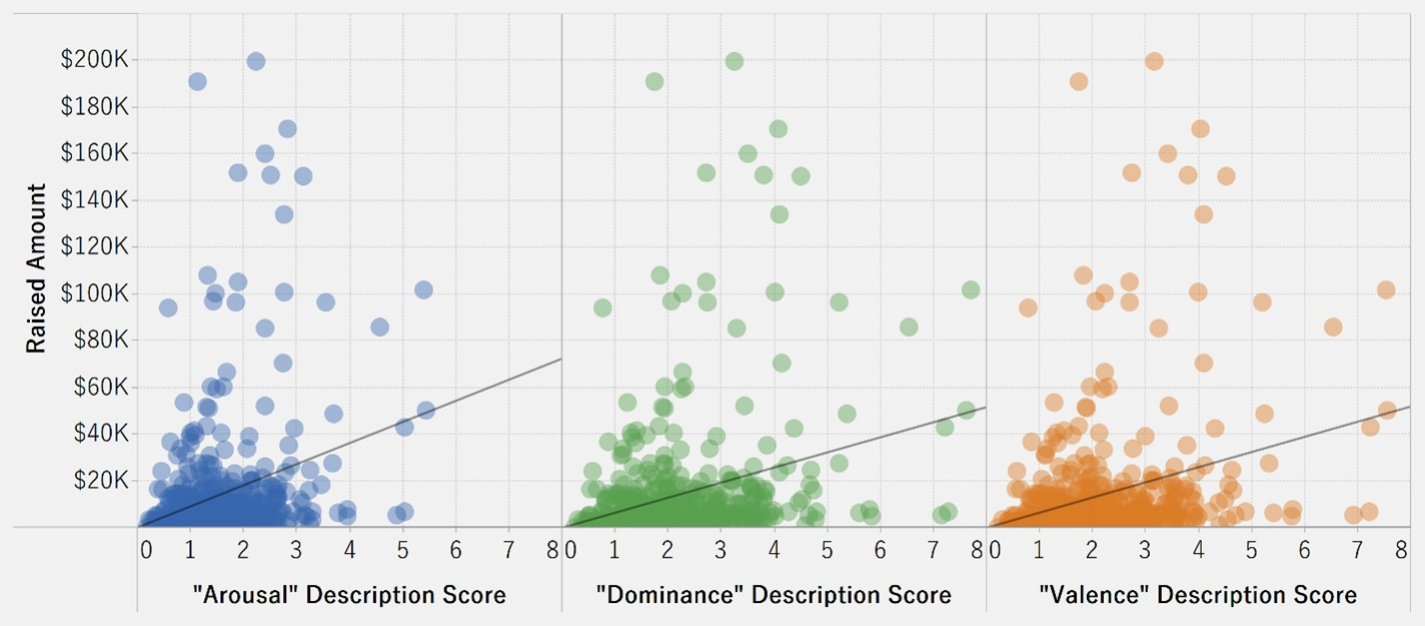
Association between three emotion variables and raised amount.

Fig 18 shows the relationship of amount raised with valence, arousal, and dominance in the case descriptions. The scores for valence, arousal, and dominance are calculated as the weighted average of the sentiment score of each word in the description. Since all the models have significant p-values (p<0.0001), the three emotion variables are significant predictors of amount raised. However, the low R-squared values indicate that the variables can predict only about 6% of the variation in amount raised. Valence, arousal, and dominance all have positive relationship with the raised amount. Therefore, high levels of valence, arousal, and dominance, as indicated in the case descriptions, are likely to increase the funding raised, but other significant factors may also need to be considered.

**Table:** 

	Valence	Arousal	Dominance
P-value	< 0.0001	< 0.0001	< 0.0001
R-Squared	0.057625	0.0555557	0.0583171

**Fig 19:**
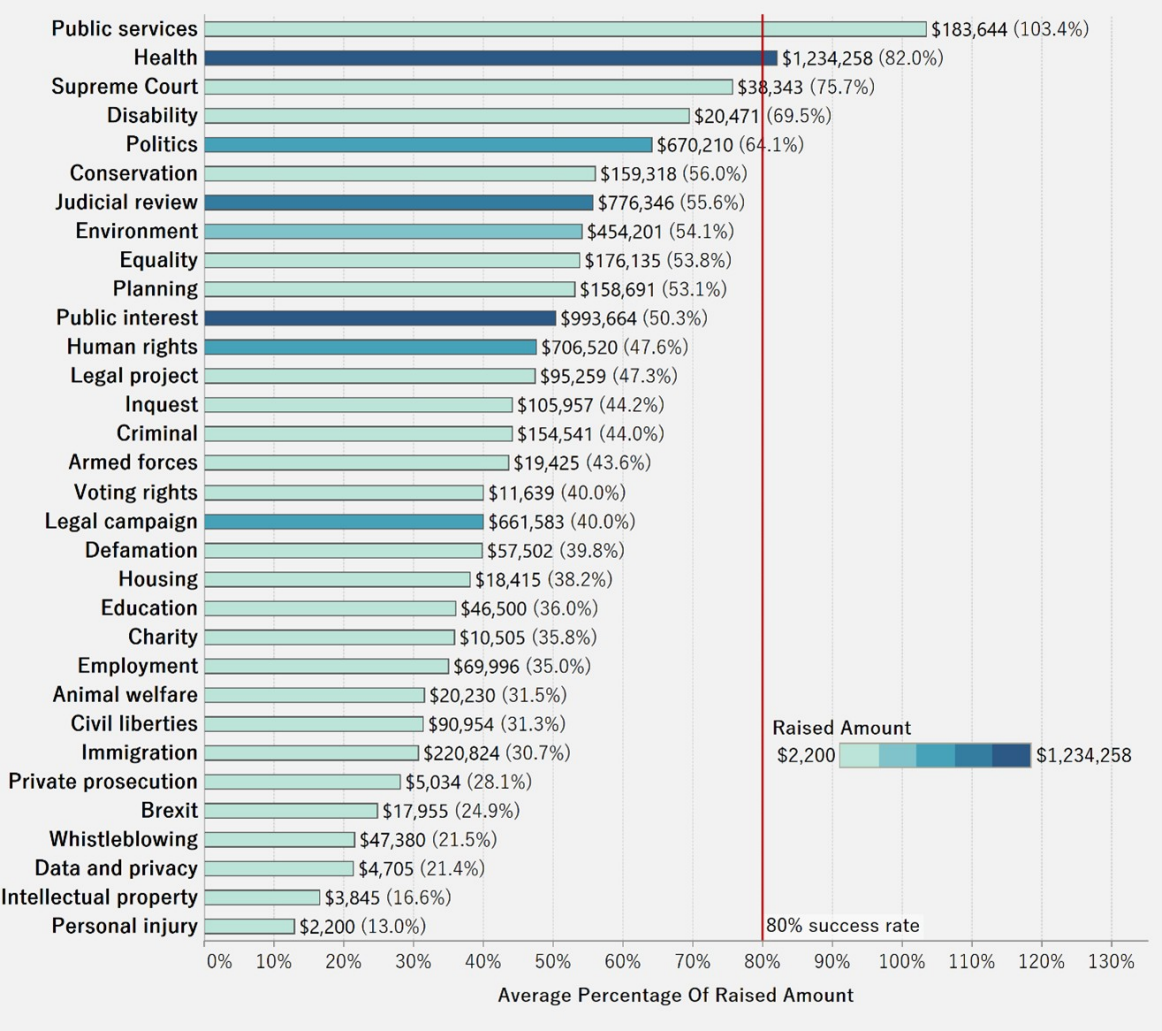
Average percent raised by funding category.

Fig 19 displays the average amount raised over the target in each category. The total amount raised is labeled and highlighted by color. Only two categories—public services and health—have an average success rate of over 80%. Cases in public services tended to raise above target levels on average, while cases in personal injury have the lowest percentage of amount raised. Though cases in health were the highest in total amount raised, cases in public services accomplished a higher percentage over the target. Donors of litigation crowdfunding appear to have a strong public awareness of the issues and tend to contribute to public services and health over private issues.

**Fig 20:**
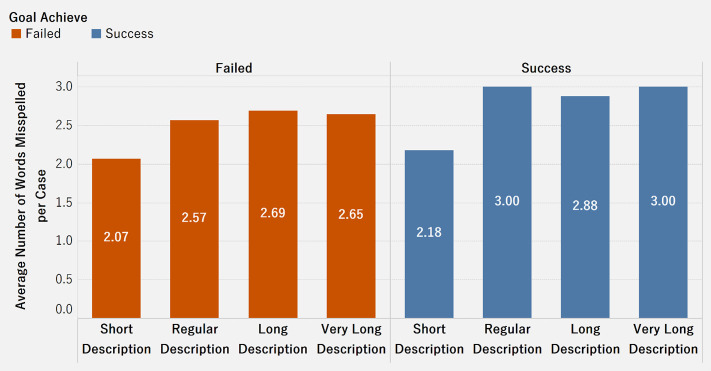
Association between misspelled words, length and goal achievement.

Fig 20 shows the average number of misspelled words for successful and failed cases, broken down by the length of case description. We considered case descriptions with 100 to 500 words as short, 500 to 1000 words as regular, 1000 words to 1500 words as long, and over 1500 words as very long. Surprisingly, successful cases have a higher average number of misspelled words than failed cases. On average, cases with regular, long, and very long descriptions have almost the same number of misspelled words. This shows that misspelled words in the case description do not appear to be significant indicators of successful or failed cases. Donors appear to be comfortable with fund seekers’ spelling errors, as long as the mistakes do not mislead in terms of the objective or the cause of the case.

**Fig 21:**
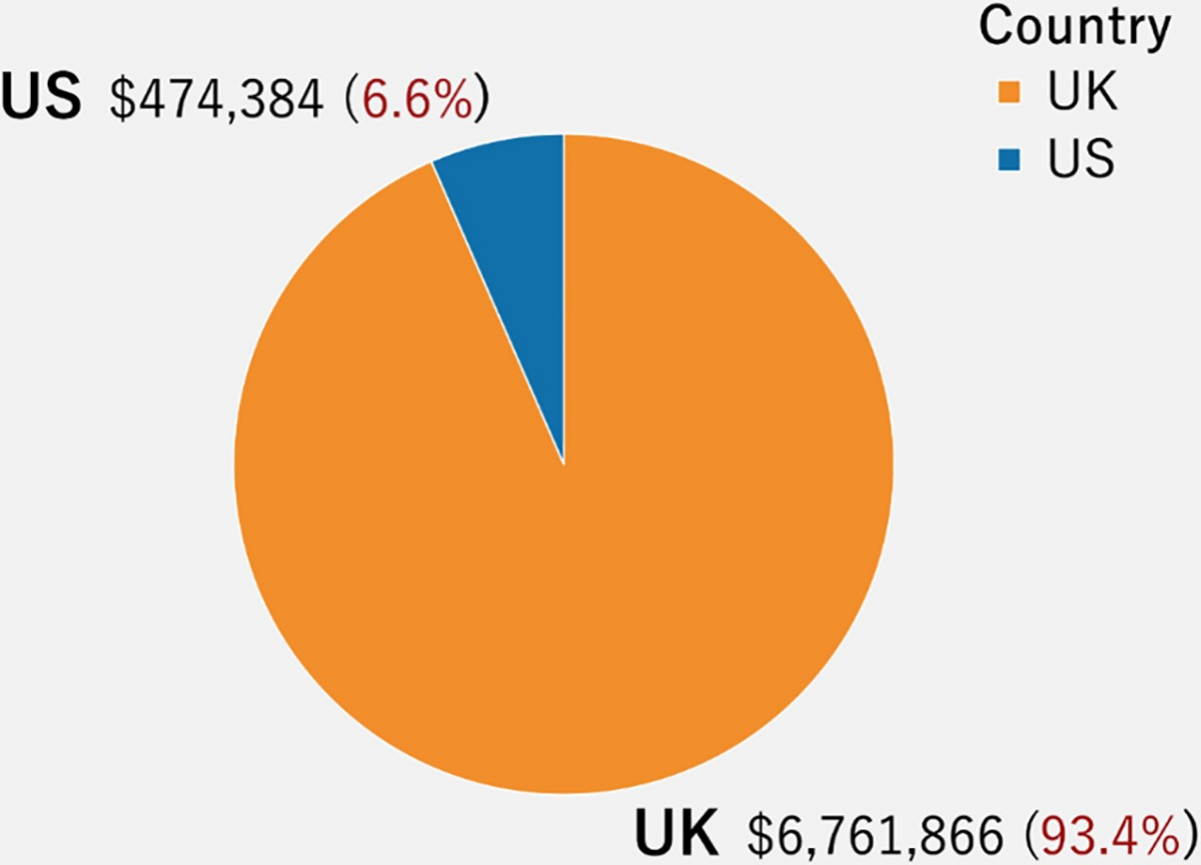
Distribution of amount raised in the U.K. and U.S.

Fig 21 compares the distribution of amount raised between the U.S and U.K. on this platform. The amount raised in the U.K., around $6 million, takes up to 93% while the amount raised in the U.S., approximately $500 thousands, is only 7%.

**Fig 22:**
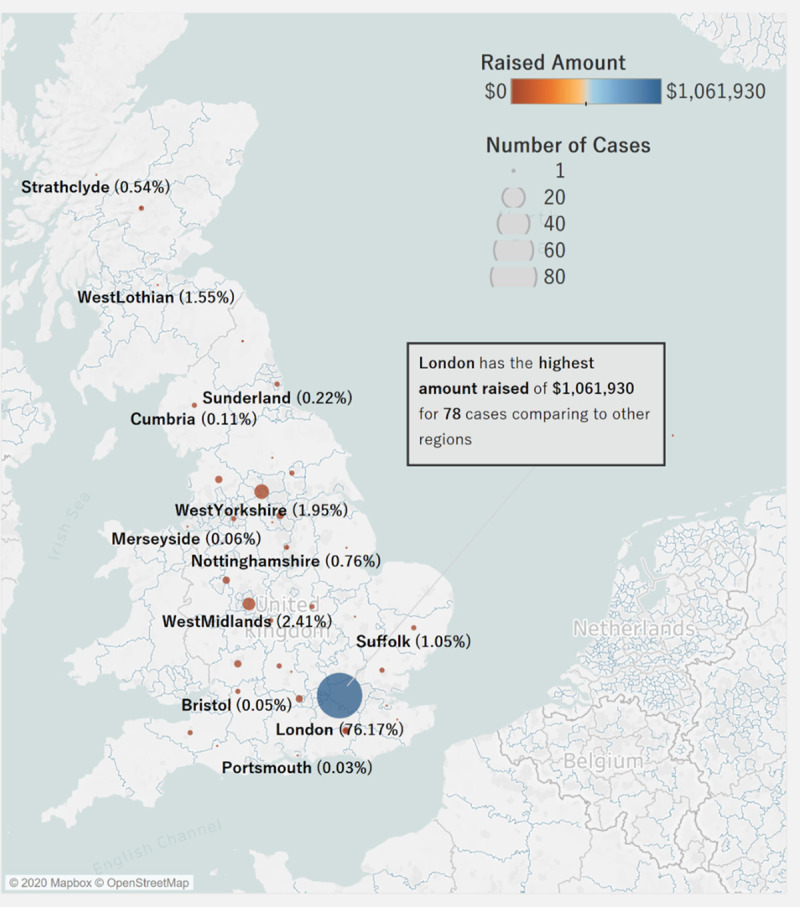
Distribution of total amount raised by Region in U.K. Visualization source: Tableau.

Fig 22 demonstrates the distribution of amount raised and the number of cases across different regions in the U.K. The map shows that London raised a significantly higher amount (around $1,061,930) with a higher number of cases compared to the other regions in the country. Most regions in the U.K. only raise from 0 to 2% for a significantly small number of cases. London is the region with the highest amount raised in the U.K.

**Fig 23:**
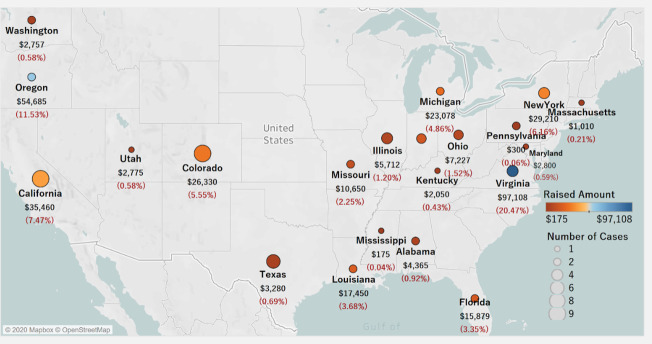
Distribution of total amount raised and number of cases by state in the U.S. Visualization Source: Tableau.

To investigate cases in the U.S., we performed a drill-down into the total raised amount and the number of cases by state (Fig 23). The color of the bubble indicates the amount raised. The size of the bubble indicates the number of crowdfunding cases in the state. Virginia is the leader in the amount raised, followed by Oregon and California. Pennsylvania and Mississippi have the lowest amount of funding. It is interesting that Virginia has raised the most, far more than states such as California and New York. The cases are mostly distributed along the east, west, and southern coast of the U.S. California and Colorado had the most litigation crowdfunding cases, but the amount raised is not significant. It is likely that, since CrowdJustice.com has just recently expanded in the U.S., the phenomenon is still too young compared to the U.K.

In comparing the regional performance between the U.S. and U.K., the American states of Virginia and Oregon follow London in terms of the amount raised. A majority of the amount raised goes to London, U.K. Crowdfunding for litigation in London is more popular than in any other area in the U.K. or the U.S. This is likely due to the fact the platform catered initially only to fund seekers in the U.K.

## Discussion

From the limited number of litigation crowdfunding cases on the CrowdJustice platform, approximately 84% are in the U.K, while about 16% are in the U.S. Likewise, 93.4% of the total amount raised is from the U.K., while 6.6% is from the U.S. Analyzing the region distribution of amount raised, the city of London individually amounts for 76% of the total funding in the U.K. (about $1,061,930), with the other cities/counties raising less than 2% of donations per location. In the U.S., Virginia and Oregon are the two states with the highest amount raised, roughly 20% and 11%, respectively. Considering the platform expanded to the U.S. only in 2017, and thus these are early days. we are likely to see more cases in the U.S. going forward., Overall, we see a higher number of cases seeking funding in the arenas of human right, environment, and judicial review.

Nevertheless, there is potential to promote the option in the other, currently less prominent, categories such as voting rights, personal injury, intellectual property, and data/privacy.

Regarding the distribution of donations, it follows that donors are willing to contribute more to cases in health, politics, and public services. It is also interesting to note that while donors are willing to contribute to education, animal welfare, data/privacy and inquest-related cases, they are unwilling to donate large amounts to same. Also, in terms of the quality of case descriptions posted on the platform, as the number of difficult words in a description increases, the amount raised also increases usually. This implies donors are likely to follow up and actually fund cases that are more professionally written. We explored the role played by the lawyer/law firm assigned for each case. More pledges follow cases that are assisted by experienced lawyers/law firms. This seems logical since donors would naturally want the cases they fund to succeed. Interestingly, health accounted for the greatest number of successful crowdfunding cases as well as for the highest amount raised. This shines a light on the general outlook that health is a critical and integral issue for living, and when donors perceive fund seekers to be struggling with health-related litigation cases, they are more likely to contribute to the case. Furthermore, we also see that the higher the number of successful cases, the higher the amount raised, relatively speaking. Another discovery is the relevance of category in the amount raised. In this regard, health and public interest cases are the most likely to succeed.

Our research analyzed the role of tone (sentiment/emotion) in the case description in terms of arousal, dominance, and valence. In this regard, all these aspects have a positive relationship with the raised amount. For example, there is a positive association between how a case description arouses interest in the case (“arousal”) and the raised amount. Crowdfunding cases will do well to keep this in mind when developing a case description. Other significant factors may also need to be considered in future research as more data becomes available. Generally, the attributes of a case description—including tone, whether the description gets updated regularly, and the length of the description—influence whether a case is funded or not. For example, when a case description is updated, there are more successful cases, at least in health, immigration, and judicial review categories. This is not the case, however, for categories such as public service, human rights, and environment. This outcome warrants further research into why such differences occur.

### Scope and limitations

Our research suffers from some limitations. We crawled the data for a specific period (2019) and therefore we offer only a snapshot of crowdfunding activity. Future studies should explore a longitudinal analysis with a larger time period and a wider range of variables. Also, while the current research deploys visual analytics methodology, it is possible for future studies to explore alternative techniques such as predictive statistical analysis and data mining (i.e., association, clustering, etc.). Considering that visual analytics methodology offers descriptive analysis, there is a need for further empirical investigation, if one wants to draw quantitative conclusions. Furthermore, while the research focuses on the relevant attributes of the crowdfunding case and the platform (such as success/failure of case, category, case description, location), it does not include the demographic information on both the fundraiser and the donor. Fundraiser or donor information can be incorporated to ascertain if certain profiles attract more funding, or if certain profiles are drawn to certain categories of cases. Additionally, specific donor information can be including in the case description to determine if the relationship between donor and fundraiser has any influence on the funding. Future studies can explore variances in the type of litigants for whom funds are raised (for example, for human rights, medical expense, dispute, etc.). Information on the insurance coverage of fundraisers is another dimension that can be incorporated, to evaluate the extent of their actual litigation cost requirement.

Also, it will be interesting to know the current situation of fundraisers as to whether the case has been successfully litigated. Finally, our sample consists of litigation cases in the U.S. and U.K. As the phenomenon of litigation crowdfunding accelerates, future studies can encompass a diverse set of cases from countries around the world.

### Conclusions and future research

This study focused on the factors and dimensions of litigation crowdfunding utilizing data from the CrowdJustice.com platform. We examined the relationships between the characteristics of a campaign and successful funding success across various categories. We also examined the nature of case descriptions and their association with funding success. We get a glimpse of the status of litigation cost and affordability dimensions through our analysis of crowdfunding campaigns across the U.K and the U.S. The research reveals the nurturing role that crowdfunding platforms can play to funding individual cases in which funding is a necessity. Typically, crowdfunding is ideal for cases related to individuals in need rather than large corporations. Our research gains significance since the topic of litigation crowdfunding is current and gaining public attention. Questions to address include whether litigation crowdfunding levels the playing field in terms of opening up financing opportunities for those individuals who cannot afford the costs of litigation. While it may support social justice, ethical concerns with regards to the kinds of campaigns must also be addressed. Most of the ethical concerns center around issues relating to both the fundraisers and donors. Examples include loss of privacy, possible fraudulence in campaigns, and the fairness and equitability in fund distribution. Debate also centers on whether campaigns can be developed to raise funds for individual versus corporate cases, and civil versus criminal cases. The increased public interest in the social dimensions of law, combined with the availability of a suite of research tools, offers rich opportunities for researchers to add considerable insight into the phenomenon of litigation crowdfunding. Further insight can accelerate the maturing process of litigation crowdfunding.

## Supporting information

S1 Dataset (CSV)

## References

[1] Belleflamme P., Lambert T., & Schwienbacher A. (2014). Crowdfunding: Tapping the right crowd. Journal of Business Venturing, 29(5), 585–609.

[2] Mollick E. (2014). The dynamics of crowdfunding: An exploratory study. Journal of Business Venturing, 29(1), 1–16.

[3] Ordanini A., Miceli L., Pizzetti M., & Parasuraman A. (2011). Crowd-funding: Transforming customers into investors through innovative service platforms. Journal of Service Management, 22(4), 443–470.

[4] Gomez M. A. (2015). Crowdfunded justice: On the potential benefits and challenges of crowdfunding as a litigation financing tool. USFL Review, 49, 307.

[5] Perry R. (2018). Crowdfunding civil justice. BCL Review, 59, 1357.

[6] Kidd J. (2011). To fund or not to fund: The need for second-best solutions to the litigation finance dilemma. Journal of Law, Economics and Policy, 8, 613.

[7] Knutsen E. S., & Walker J. (2010). The costs and funding of civil litigation: A comparative perspective. New York, NY: Bloomsbury Publishing.

[8] Steinitz M., & Field A. C. (2013). A model litigation finance contract. Iowa Law Review, 99, 711.

[9] Feldman D. (1992). Public interest litigation and constitutional theory in comparative perspective. Modern Law Review, 55, 44.

[10] Skiba P. M., & Xiao J. (2017). Consumer litigation funding: Just another form of payday lending. Law & Contemporary Problems, 80, 117.

[11] American Bar Association. (2018). A lawyer’s obligations when clients use companies or brokers to finance the lawyer’s fee. Retrieved from https://www.americanbar.org/content/dam/aba/images/news/2018/11/formal_opin_484.pdf

[12] British Columbia Law Institute. (2017). Study paper on financing litigation. Retrieved from https://s3.amazonaws.com/tld-documents.llnassets.com/0005000/5299/2017-10-04-bcli-study-paper-on-financing-litigation-publication-copy-rev.pdf

[13] Cheung K. (2013). Considering crowdfunding as a litigation aid? You should. FindLaw. Retrieved from https://blogs.findlaw.com/in_house/2013/05/considered-crowdfunding-a-business-litigation-strategy-you-should.html

[14] Zaleska-Korziuk K. (2018). When the good samaritan pays: The phenomenon of strategic third-party funding. Asper Review of International Business and Trade Law, 18, 160.

[15] André K., Bureau S., Gautier A., & Rubel O. (2017). Beyond the opposition between altruism and self-interest: Reciprocal giving in reward-based crowdfunding. Journal of Business Ethics, 146(2), 313–332.

[16] NeJaime D. (2013). The view from below: Public interest lawyering, social change, and adjudication. UCLA Law Review Discourse, 61, 182.

[17] Kappel T. (2008). Ex ante crowdfunding and the recording industry: A model for the US. Loyola of Los Angeles Entertainment Law Review, 29, 375.

[18] Garber S. (2010). Alternative litigation financing in the United States. RAND Corporation.

[19] Cumming D.J., Johan S.A. & Zhang Y. (2019). The Role of Due Diligence in Crowdfunding Platforms. Journal of Banking & Finance, 108(105661). 10.1016/j.jbankfin.2019.105661

[20] Cumming D.J. & Zambelli S. (2017). Due diligence and investee performance. European Journal of Financial Management 23, 211–253.

[21] Yung C. (2009). Entrepreneurial financing and costly due diligence. Financial Review 44, 137–149.

[22] Avraham R., & Sebok A. J. (2018). An empirical investigation of third party consumer litigant funding. Cornell Law Review.

[23] British Columbia Law Institute & Canadian Centre for Elder Law. (2017). Annual report. British Columbia Law Institute.

[24] Elliot M. (2016). Trial by social-media: The rise of litigation crowdfunding. University of Cincinnati Law Review, 84, 529.

[25] Graham M. (2015). Funded justice aims to help people raise funds for legal fees. Chicago Tribune. Retrieved from https://www.chicagotribune.com/business/blue-sky/chi-funded-justice-michael-helfand-bsi-20150105-story.html

[26] Rozenberg J. (2015). Is crowdfunded litigation the future of justice? The Guardian. Retrieved from https://www.theguardian.com/commentisfree/2015/may/25/crowdfunded-litigation-future-justice-crowdjustice

[27] Avraham R., & Wickelgren A. (2013). Third-party litigation funding-A signaling model. DePaul Law Review, 63, 233.

[28] Bayer M., & Schacht A. (2014). Event-related brain responses to emotional words, pictures, and faces–A cross-domain comparison. Frontiers in Psychology, 5, 1106. 10.3389/fpsyg.2014.01106 25339927 PMC4186271

[29] Beall P. M., & Herbert A. M. (2008). The face wins: Stronger automatic processing of affect in facial expressions than words in a modified Stroop task. Cognition and Emotion, 22(8), 1613–1642.

[30] Chakhar S., Ishizaka A., Thorpe A., Cox J., Nguyen T., & Ford L. (2020). Calculating the relative importance of condition attributes based on the characteristics of decision rules and attribute reducts: application to crowdfunding. European Journal of Operational Research, 286, 689–712.3

[31] Kensinger E. A., & Schacter D. L. (2006). Processing emotional pictures and words: Effects of valence and arousal. Cognitive, Affective, & Behavioral Neuroscience, 6(2), 110–126. 10.3758/cabn.6.2.110 17007232

[32] Killgore W. D. S. (1999). Affective valence and arousal in self-rated depression and anxiety. Perceptual and Motor Skills, 89(1), 301–304. 10.2466/pms.1999.89.1.301 10544430

[33] Larsen R. J., & Diener E. (1992). Promises and problems with the circumplex model of emotion. In Clark M. S. (Ed.), Review of personality and social psychology, No. 13. Emotion (pp. 25–59). Thousand Oaks, CA: Sage Publications, Inc.

[34] Mäntylä, M., Adams, B., Destefanis, G., Graziotin, D., & Ortu, M. (2016). Mining valence, arousal, and dominance: Possibilities for detecting burnout and productivity? In Proceedings of the 13th International Conference on Mining Software Repositories (pp. 247–258). ACM.

[35] Oosterhof N. N., & Todorov A. (2008). The functional basis of face evaluation. Proceedings of the National Academy of Sciences, 105(32), 11087–11092. 10.1073/pnas.0805664105 18685089 PMC2516255

[36] Ovaysikia S., Chan J. L., Tahir K., & DeSouza J. F. X. (2011). Word wins over face: emotional Stroop effect activates the frontal cortical network. Frontiers in human neuroscience, 4, 234. 10.3389/fnhum.2010.00234 21258644 PMC3020489

[37] Russell J. A. (1980). A circumplex model of affect. Journal of Personality and Social Psychology, 39(6), 1161. 10.1037//0022-3514.79.2.286 10948981

[38] Sutton T. M., Herbert A. M., & Clark D. Q. (2019). Valence, arousal, and dominance ratings for facial stimuli. Quarterly Journal of Experimental Psychology. 10.1177/1747021818770316 30760113

[39] Watson D., & Tellegen A. (1985). Toward a consensual structure of mood. Psychological Bulletin, 98(2), 219. 10.1037//0033-2909.98.2.219 3901060

[40] Barsade S. G., & Gibson D. E. (2007). Why does affect matter in organizations? Academy of Management Perspectives, 21(1), 36–59.

[41] Graziotin D., Wang X., & Abrahamsson P. (2015a). Do feelings matter? On the correlation of affects and the self‐assessed productivity in software engineering. Journal of Software: Evolution and Process, 27(7), 467–487.

[42] Khan I. A., Brinkman W.-P., & Hierons R. M. (2011). Do moods affect programmers’ debug performance? Cognition, Technology & Work, 13(4), 245–258.

[43] Börner K., Bueckle A., & Ginda M. (2019). Data visualization literacy: Definitions, conceptual frameworks, exercises, and assessments. Proceedings of the National Academy of Sciences, 116(6), 1857–1864. 10.1073/pnas.1807180116 PMC6369751 30718386

[44] Keim, D., Kohlhammer, J., Ellis, G., & Mansman, F. (2010). Solving Problems with Visual Analytics. Retrieved from http://www.vismaster.eu/wp-content/uploads/2010/11/VisMaster-book-lowres.pdf

[45] Keim D.A. (2001). Visual exploration of large data sets. Communications of the ACM, 44, 38–44.

[46] Wong P.C., & Thomas J. (2004). Visual analytics—Guest editors’ introduction. IEEE Transactions on Computer Graphics and Applications, 24, 20–21. 10.1109/mcg.2004.39 15628096

[47] Kohlhammer J, Keim D, Pohl M, Santucci G, Andrienko G. (2011). Solving problems with visual analytics. Procedia Comput Sci 2011;7:117–120 [FREE Full text] 10.1016/j.procs.2011.12.035 76.

[48] Thomas J., & Cook K. (2005). Illuminating the Path: Research and Department Agenda for Visual Analytics. United States Department of Homeland Security: Washington, DC.

[49] Warriner A. B., Kuperman V., & Brysbaert M. (2013). Norms of valence, arousal, and dominance for 13,915 English lemmas. Behavior research methods, 45(4), 1191–1207. 10.3758/s13428-012-0314-x 23404613

[50] Li F. (2008). Annual report readabililty, current earnings, and earnings persistence. Journal of Accounting and Economics 45, 221–247.

